# Hoarding titmice predominantly use Familiarity, and not Recollection, when remembering cache locations

**DOI:** 10.1007/s10071-023-01829-3

**Published:** 2023-10-21

**Authors:** Tom V. Smulders, Laura J. Douglas, Daniel Reza, Lucinda H. Male, Alexander Prysce, Amélie Alix, Alexander de Guzman Dodd, Jenny C. A. Read

**Affiliations:** 1 Centre for Behaviour & Evolution and Biosciences Institute, Newcastle University, Newcastle upon Tyne, NE2 4HH UK; 2 School of Psychology, Newcastle University, Newcastle upon Tyne, NE2 4DR UK

**Keywords:** Comparative cognition, Episodic-like memory, Scatter-hoarding, Cognitive evolution, Hippocampus, *Periparus ater*

## Abstract

Scatter-hoarding birds find their caches using spatial memory and have an enlarged hippocampus. Finding a cache site could be achieved using either Recollection (a discrete recalling of previously experienced information) or Familiarity (a feeling of “having encountered something before”). In humans, these two processes can be distinguished using receiver operating characteristic (ROC) curves. ROC curves for olfactory memory in rats have shown the hippocampus is involved in Recollection, but not Familiarity. We test the hypothesis that food-hoarding birds, having a larger hippocampus, primarily use Recollection to find their caches. We validate a novel method of constructing ROC curves in humans and apply this method to cache retrieval by coal tits (*Periparus ater*). Both humans and birds mainly use Familiarity in finding their caches, with lower contribution of Recollection. This contribution is not significantly different from chance in birds, but a small contribution cannot be ruled out. Memory performance decreases with increasing retention interval in birds. The ecology of food-hoarding Parids makes it plausible that they mainly use Familiarity in the memory for caches. The larger hippocampus could be related to associating cache contents and temporal context with cache locations, rather than Recollection of the spatial information itself.

**Supplementary Information:**

The online version contains supplementary material available at 10.1007/s10071-023-01829-3.

## Background

Food-hoarding chickadees and titmice are scatter-hoarders that hide virtually every food item in a different location. To be more likely to retrieve these items than other conspecifics, these birds use spatial memory to relocate their cache sites (Cowie, Krebs and Sherry 1981; Sherry 1982, 1984a, 1984b; Sherry, Krebs and Cowie 1981). This memory has been shown to last for at least 4–6 weeks, both in the lab (Brodin and Kunz 1997; Male and Smulders 2007a) and in the field (Brodin 1994), and is used not only to retrieve their caches, but also to avoid caching too close to existing cache sites (Male and Smulders 2007b). Because these birds are so reliant on spatial memory, it has been hypothesized that they have an adaptively specialized spatial memory system (Brodin and Bolhuis 2008; Krebs 1990; Shettleworth 2003). Evidence for this claim comes from the fact that, within the same species, birds from populations that live in harsher climates perform better in cache retrieval trials in captivity (Pravosudov and Clayton 2002). Birds from these same populations also have a larger hippocampal formation, known to be required for successful cache retrieval, than birds from less harsh environments (Pravosudov and Clayton 2002; Roth, la Dage, Freas and Pravosudov 2012; Roth and Pravosudov 2009). Comparing across species, scatter-hoarding birds also have a larger hippocampal formation than non-hoarding birds (Krebs, Sherry, Healy, Perry and Vaccarino 1989; Sherry, Vaccarino, Buckenham, and Herz, 1989), although attempts to link hippocampal volume to degree of hoarding specialization have met with mixed results (Brodin and Lundborg 2003; Garamszegi and Lucas 2005; Hampton, Sherry, Shettleworth, Khurgel, and Ivy 1995; Healy, Clayton, and Krebs 1994; Healy and Krebs 1992; Lucas, Brodin, de Kort, and Clayton 2004).

Although it seems well established that scatter-hoarding birds have an adaptive specialization in spatial memory and the associated neural structures, the nature of this specialization is still unclear. In direct inter-species comparisons, scatter-hoarding birds are more biased toward using spatial information, rather than local (color and shape) information, when the two sets of cues are pitched against each other (Clayton and Krebs 1994). Food-hoarding birds have more accurate and longer-lasting working memory in a spatial delayed matching to sample task than non-hoarding birds (Biegler, McGregor, Krebs, and Healy 2001; McGregor and Healy 1999). Additionally, food-hoarding birds have been shown not only to remember the locations of cache sites, but also their content, and the time that has elapsed since the cache was made (Clayton and Dickinson 1998; Clayton, Yu, and Dickinson 2003; Feeney, Roberts, and Sherry 2009, 2011; Sherry 1984a; Zinkivskay, Nazir, and Smulders 2009). This integrated memory has been likened to episodic memory in humans (Clayton and Dickinson 1998). Episodic memory involves the hippocampus (Yonelinas 2002; Yonelinas, Kroll, Dobbins, Lazzara, and Knight 1998), and therefore the enlarged hippocampal formation in food-hoarding birds might well be associated with improved episodic(-like) memory. In humans, episodic memory is associated with free recall of information (also called Recollection) (Yonelinas 2001). This is often contrasted with Familiarity, which is a different memory process that relies on being exposed to a stimulus which then triggers a feeling of “having seen this before”. In humans, Recollection is seen to decline quickly in the first 24h, and gradually after that (Gardiner and Java 1991). Familiarity declines slowly from the start (Gardiner and Java 1991), and may not change significantly for up to 2 weeks, at least when the information to be retained is about properties of artificial grammar (Tunney 2010; Tunney and Bezzina 2007).

An adaptive specialization in episodic memory, as evidenced by the enlarged hippocampal formation, would therefore be predicted to be associated with strong use of Recollection over Familiarity. But how can we distinguish between memory based on Recollection and memory based on Familiarity? One method that has been used in both humans and other animals, involves Receiver Operating Characteristic (ROC) curves (Yonelinas 1994; Yonelinas, Dobbins, Szymanski, Dhaliwal, and King 1996). ROC curves are constructed by grouping the responses by the confidence level with which these responses are made. Human participants are asked to rate the confidence levels of their decisions (from “certain” to “guessing”). This is not an option in non-human animals. To obtain such levels of confidence in a non-verbal manner, previous studies have used methods based on manipulating costs and benefits of making a “old” or a “new” response (Fortin, Wright, and Eichenbaum 2004; Guderian, Brigham, and Mishkin 2011). The assumption behind this approach is that when the cost of a “false alarm” (i.e., responding “old” when the item is in fact “new”) is high, animals will only make the “old” response if they are highly confident of their memory. Others have used response latency (Weidemann and Kahana 2016), on the grounds that responses based on higher confidence will be made more quickly. Both approaches are based on the assumption that animals have a form of metacognition that allows them to access the reliability of their memory traces in some way. This has been shown in, e.g., macaques (*Macaca mulatta*; Hampton 2001), large-billed crows (*Corvus macrorhynchos*; Goto and Watanabe 2012), pigeons (*Columba livia*; Adams and Santi 2011), and even bees (*Apis mellifera*; Perry and Barron 2013). Arbilly and Lotem (2017) have made a strong argument for why such an ability does not need to involve highly complex cognition. If animals can perceive the reliability of memory traces and act on it, then they act as humans who would have higher or lower confidence in a particular memory trace.

However, manipulating costs and benefits to obtain ROC curves requires extensive training, and the use of latencies requires discrete trials that have a clear starting time. Birds retrieving their caches perform spontaneous behaviors without a clear start to each “trial” (retrieval). Previous methods are, therefore, not applicable. We decided to take a different approach. It is possible that animals (and humans), when given a choice of stimuli to respond to, will respond first to more reliable memory traces, before acting on less reliable ones. It has indeed been shown that food-hoarding birds perform better on early decisions than later decisions (Kamil and Balda 1990), indicating that the order in which cache sites are searched may reflect how “confident” the bird is of the food’s location.

In the current study, we first explore whether the temporal order in which decisions are made can indeed act as a proxy for the confidence level of those decisions. We report a real-world spatial memory experiment with humans, designed to match the experience of the birds as much as possible, using a large space relative to the individuals and retrieving cached items after a one-day retention interval. We find that results using actual self-reported confidence are similar to those obtained by using decision order as a proxy for confidence. Having, thus, demonstrated that decision order is a valid proxy for confidence in humans, we then make the assumption that a similar relationship holds in coal tits, and use this to reanalyze existing data obtained from coal tits (*Periparus ater*) retrieving their caches over different retention intervals (Male and Smulders 2007a). Our prediction is that coal tits will rely predominantly on Recollection processes in recalling their cache sites, and that this reliance on Recollection will diminish with increasing retention intervals.

## Methods

### Subjects

#### Coal tits

The coal tit data are a reanalysis of data published in a previous paper (Male and Smulders 2007a). Nine coal tits of unknown sex (4 juveniles, 5 adults) were captured in Northumberland in September 2004 under English Nature license number 20042059. The birds were caught by a qualified ringer using mist nets on private land and were transported in cotton holding bags in which they spent a maximum of three hours. The coal tits’ ages were determined from the moulting patterns of their greater coverts (Svensson 1992). In April 2005, we released all the birds in the same area in which they were caught. All animal experimentation was done according to the ASAB/ABS guidelines and within the law of the United Kingdom. The birds maintained their weight and health during captivity. Reanalysis of existing data reduces the use of animals, one of the 3Rs.

#### Humans

54 participants were recruited in June–August 2022 (28M/26F, 19–66 years (*Median* = 25.00, *IQR* = 5)) via advertisements placed on DR’s personal accounts on social media platforms (i.e., Facebook, Instagram, Twitter) and via emails distributed to Newcastle University staff and students. The study was also advertised using posters displayed around Newcastle University campus, as well as on an undergraduate psychology online learning platform through which students could participate in return for research participation credits. To register for the study, participants were asked to contact DR in order to schedule two time-slots across two consecutive days. All participants were rewarded with a £10 Amazon gift voucher upon completion of both parts of the study. Participants had to be over the age of 18 years and not knowingly suffer from a memory impairment. Ethics was approved by the Newcastle University Ethics Committee (Ref: 23297/2022).

### Procedure

#### Coal tits

We ran the experiment from December 2004 to April 2005. The birds were maintained on a 8.5h:15.5h Light:Dark cycle and at a temperature of between 14°C and 19°C. During the experiments, the birds were housed individually in cages which measured 85.0 × 45.5 × 95.0 cm (width × depth × height) in a room adjacent to the experimental aviary. Water was available ad libitum. The coal tits were fed a daily diet of four split peanuts, two sunflower seeds, three pine nuts, two wax worms, four mealworms, and one scoop of EMP/Universal bird mix. They were deprived of food for one hour before each storage, retrieval, or foraging session. We tested the birds in an experimental aviary measuring 216 × 350 × 235 cm (width × depth × height), while viewing through a one-way observation window from an observation room. Water was available on a platform in the center of the experimental aviary. 84–86 storage sites (depending on the room layout) were available in large tree branches (8–13 per branch), placed upright in concrete blocks and in 53 wooden blocks suspended in three concentric rings from the ceiling of the aviary. Each storage site consisted of a hole of 0.5 cm in diameter and 1.0 cm in depth, with a 5-cm-long perch below it. The holes were obscured with lengths of thick string which allowed the bird access but restricted its view of the hole. Once a bird checked a hole, the string was pushed out of the way, effectively marking the location as having been checked. There was colored tape below each storage site to help the observer identify the location. Colors were not unique to each cache site. Spruce branches were randomly secured on nine of the wooden blocks to act as spatial landmarks. In addition, other landmarks included colored pieces of cardboard which were positioned on the aviary walls and various objects which were suspended from the ceiling. The birds were tested in two different aviary layouts, which consisted of three trees in different positions, different locations for the spruce branches, and different types of cardboard and objects. These made the two aviary layouts as distinct as possible.

The birds were habituated to the experimental aviary by allowing them to forage and eat while in the room. They received daily training sessions for a two-week period until they were readily flying from the housing cage to the experimental aviary and back again when we turned off the lights. Each coal tit was given six 30-min storage sessions in the aviary and retrieved these caches in a 4-min retrieval session after six different retention intervals: 1, 3, 7, 14, 28, or 42 days. These sessions constitute the “Retrieval” condition.

We split the coal tits into two subgroups (A and B) of approximately the same number of adults and juveniles. Group A birds hoarded food in aviary layout 1 and Group B birds hoarded in aviary layout 2. The order of the retention intervals tested was randomized at the start (a different random order for each of the two groups of birds) and all birds within the same group were then tested in that same random order. After all Retrieval trials had finished, Group A birds, which cached in aviary layout 1, were given the opportunity to forage in aviary layout 2 on cache distributions that replicated those made by Group B birds during their hoarding sessions, and vice versa. These foraging sessions constitute the “Naïve” condition. Although “Naïve” sessions do not themselves have a retention interval, they were labeled with the retention interval used for the same distribution in the Retrieval condition to match them in the analysis.

The coal tits, therefore, foraged for conspecific coal tits’ caches in a different aviary layout to where they had themselves hoarded food previously. This was to minimize the possibility that any remaining memories for cache locations would interfere with the foraging of birds in the Naïve condition. If any bird hoarded food during the Naïve foraging session, it was given an additional session to retrieve this food. This was to minimize the risk that the memory of their own hoarded food would interfere with future foraging sessions. To allow for age-related differences in caching preferences, adult coal tits foraged for other adult coal tits’ caches and juvenile coal tits foraged for other juvenile coal tits’ caches.

It is possible that birds learn about the kinds of locations that are more likely to have seeds hidden in them, and therefore improve with repeated “Naïve” foraging sessions. To maximize our ability to detect above chance memory performance at the 42-day retention interval, we needed to compare that to truly naïve birds, rather than birds that had been trained on a few distributions already. The birds in the Naïve condition would really only be totally naïve to the distributions made by other birds (i.e., have no experience of where food can be found in the aviary layout that is different from the one in which they themselves hoarded) on the very first Naïve foraging trial. We, therefore, matched the first Naïve foraging trial to the 42-day retention interval of the birds on whose distributions they were foraging. The subsequent Naïve foraging trials were then matched to the Retrieval trials (i.e., used the distributions generated by a bird from the opposite group in the “Retrieval” condition) in descending order of the original retention intervals (which themselves had been conducted in a random order). Data were extracted from paper records of the birds’ movements through the room and the different cache locations checked, as recorded by LHM.

#### Humans

All sessions were conducted a large, tiered lecture theater (20m (w) x 15m (d) × 3.7m (h)), containing a total of 318 seats and desks, distributed across 9 tiered rows. A total of 84 film canisters (lids closed) were distributed across the chairs, tables and floor of the lecture theater in a pseudo-randomized, but close to uniform distribution (Fig.S1), to replicate the cache sites of the bird study. Canisters were placed onto the surfaces without attaching them and were approximately 1–2m away from the nearest neighbor. Each canister was identifiable to the researcher through a barcode and number that was attached to the inside of the film canister lid.

The 54 participants were randomly assigned to one of two experimental groups: in the Choice group, participants hid coins in their own chosen locations (like the birds), while in the Random group, participants hid coins in locations indicated by the researcher. We decided to include the Random group in this study, as human participants in the Choice group might use systematic means to remember their coin locations (e.g., “the third canister in each row”), severely simplifying the memory task. To generate the coin locations of participants in the Random group, a random number generator was used in MS Excel® which generated the required numbers between 1 and 84. Each person in the Random group received a different random distribution. To determine how many coins they needed to hide, each participant was matched to one of the birds in the 1-day retention interval and was asked to hide the same number of items. Since we had six times as many human participants as birds, three human participants in each group were matched with every bird. Based on this match, in each group, three participants hid 4 coins each, three hid 6 coins each, three hid 8 coins each, three hid 9 coins each, three hid 10 coins each, six hid 12 coins each, and six hid 13 coins each (see the Data Accessibility statement for access to the raw data).

Participants were given instructions and provided with an information sheet and consent agreement before entering the room. They were instructed to count under their breath while hiding and retrieving, in an attempt to suppress verbal encoding of the locations. On Day 1, participants were asked to walk around the lecture theater to familiarize themselves with the locations of the film canisters around the room. Once ready, participants took the first £1 coin from a paper plate placed between locations 78 and 79 (see Supplementary Materials for a map) and hid it in one of the film canisters that were placed around the room. They repeated this until all the coins had been hidden. After opening and inserting each £1 coin into a canister, participants handed over the film lid to the researcher, who proceeded to scan the barcode and return the film canister for the participant to close. The scanner recorded the ID of the film canister directly to a spreadsheet, which added a timestamp to each site location. Participants in the Choice group were instructed to distribute the coins in such a way not only that they would be able to remember where they put them, but also that they would lose as few as possible to a naïve person looking for their coins. Participants in the Random group hid their £1 coins in randomly generated film canister locations pointed to, in silence, by the researcher.

On Day 2, prior to the arrival of each participant, the researcher redistributed each participant’s unique coin distribution ready for the participant to retrieve. Upon arrival, participants were reminded of the instructions. Participants were asked to find the coins they had hidden the previous day, making as few mistakes as possible, by going to the canisters and opening them. For each chosen canister, they provided a confidence rating (“sure”, “probable”, or “guess”) prior to touching that canister. After opening each canister, the researcher scanned the barcode to record the location ID, confidence rating, and timestamp. Each canister that had been checked remained with its lid off next to where it was located, to mark the location as checked, similar to what the birds experienced. Participants who hid 10 or fewer coins had a total of 20 attempts to look for all of their coins, while participants who hid more than 10 coins had a total of 30 attempts. We imposed these maxima to prevent participants from using a strategy of systematically searching through the canisters. The ROC analysis would never use more than twice the number of trials as there are coins (see later), so there was also no need to collect more data. If a participant exhausted all of their attempts and did not successfully find all of the hidden coins, the researcher proceeded to show the participant where the remaining coins were hidden. This dataset represented the Retrieval condition for this participant. ROC curves would then be constructed in two ways: using the self-reported confidence levels and using the order of choices (see below).

Once finished, the participant was asked to step out of the room while the researcher prepared the film canisters for the Naïve condition search, by hiding the same number of coins as this participant had hidden, but in locations used by another participant. Due to practical constraints, we were not able to use a different room layout for two subgroups of participants as we did for the birds. Individuals in the Choice group searched for the coin distributions from the Random group, and vice versa. Both sets of participants were informed that another person had hidden these coins with the aim of retrieving them, but also with the aim of preventing others from finding them. Participants were given a total of 40 attempts to retrieve all the coins. Data were collected as before, but without confidence scores. All checked film canisters remained with their lids off. This dataset represented the Naïve condition for the matched participant. Since confidence values were not collected (as they would have been meaningless as measures of memory), ROC curves could be constructed only using the order of choices. Upon completion of the experiment, participants were verbally debriefed and handed a printed debrief letter.

## Quantification and statistical analysis

### Data extraction

For human participants, data were collected as described above, and for the coal tits, from written observation records. Trials in which fewer than 4 seeds were cached were not used in this reanalysis. We recorded the order in which participants and birds visited the different locations, indicating when a location where the bird or participant uncovered the hole/canister was correct and when it was not (false alarm). In addition, for the human participants, we also recorded their level of confidence (self-report, 3 levels: “sure”, “probable”, or “guess”), as expressed during the retrieval phase. Revisits were ignored. These records are publicly available at the link provided in the Data Accessibility statement.

For each participant or bird, we determined how many items were hidden in a given trial (target items, so this number is *N*_tgt_). We then went through the decisions made in the Retrieval or Naïve trials, starting from the first location that was investigated. We only included decisions into the ROC analysis up to and including the *N*_tgt_^th^ false alarm. This means all the other locations were treated as misses (if they were target locations, but had not been searched by then) or correct rejects (all other locations) rather than hits/false alarms. For example, if a participant had *N*_tgt_ = 12 items to find, we would only analyze their decisions up to when they make their 12^th^ false alarm (incorrect) decision.

### Memory model

In classic memory models developed by Yonelinas (Yonelinas 1994, 2001; Yonelinas et al. 1996), targets are remembered if *either* (a) they are Recollected (probability *R*), *or* (b) they trigger a sense of Familiarity exceeding a given decision criterion *C*. We assume that on each trial the sense of Familiarity, *f,* is drawn from a Gaussian distribution with a mean of μ for locations where items were hidden and a mean of 0 for locations where items have not been hidden, with the same SD (σ) in both cases. The ratio μ/σ is the discriminability index d’, while for convenience we write the normalized decision criterion C/σ as c. Thus, the “hit” rate for targets is:

1 $${P}_{Hit}=R+\left(1-R\right)\frac{1}{\sigma \sqrt{2\pi }}{\int }_{C}^{\infty }df{e}^{-\frac{{\left(f-\mu \right)}^{2}}{2{\sigma }^{2}}}=R+\left(1-R\right)\left[1-\Phi \left(\frac{C-\mu }{\sigma }\right)\right]=R+\left(1-R\right)\Phi \left({d}^{\mathrm{^{\prime}}}-c\right).$$

While the “false alarm” rate for lures is

2 $${P}_{FA}=\frac{1}{\sigma \sqrt{2\pi }}{\int }_{C}^{\infty }df{e}^{-\frac{{f}^{2}}{2{\sigma }^{2}}}=1-\Phi \left(\frac{C}{\sigma }\right)=\Phi \left(-c\right),$$

where Φ represents the cumulative standard Gaussian, and we have used the fact that 1-Φ(*x*) = Φ(-*x*).

The classic model assumes that a particular individual in a particular session has fixed values of R, d’ and c. These three parameters cannot be inferred from observing the two variables* P*_hit_ and *P*_FA_. However, if we could re-run the experiment and change the normalized decision criterion *c*, without changing R or d’, we would obtain a new pair of *P*_hit_ and *P*_FA_, and this additional information would in theory enable us to recover R and d’.

**Fig. 1:**
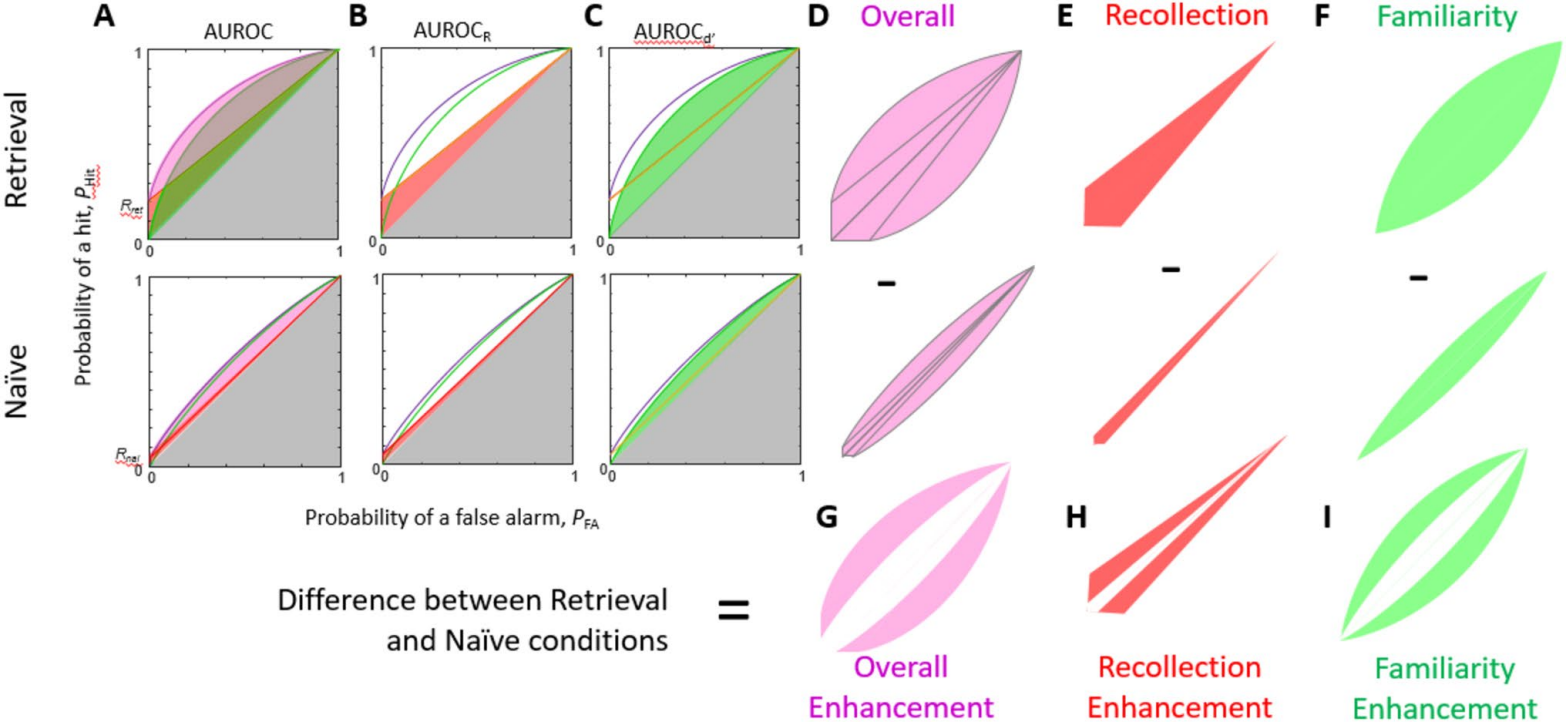
Graphical illustration of our memory metrics. **A**: ROC curves in Retrieval (upper panel) and Naïve (lower panel) conditions. The area under the ROC curve, the AUROC, is shaded. To convert AUROC to a measure of Overall performance, we subtract the area corresponding to performance less than chance, shaded in gray, and double the remainder. This is shown as the pink region in D. **B**: The shaded region is AUROC_R_, i.e., the area under the ROC curve which would be obtained with the same value of R (same intercept) but d’ = 0 (no curvature). To obtain a measure of Recollection, we again subtract the area below chance and double the remainder (red region in **E**). This is in fact equal to the parameter R. **C**: The shaded region is AUROC_d’_, i.e., the area under the ROC curve which would be obtained with the same value of d’ but no recollection. To obtain a measure of Familiarity, we again subtract the area below chance and double the remainder (green region in **F**). We do this for the Retrieval and Naïve conditions. The difference between the two conditions gives us our measure of enhancement: the enhancement in Overall performance (**G**), in Recollection specifically (**H**) and in Familiarity specifically (**I**)

The plot of *P*_hit_ against *P*_FA_ as *c* varies is called the receiver operating characteristic (ROC) curve. At the extreme, the ROC curve begins at the top right, *P*_hit_ = *P*_FA_ = 1 for very low, negative values of the normalized decision criterion *c* (i.e., when even the lowest strength of feeling of familiarity is sufficient to trigger investigation), and ends at the bottom left* P*_hit_ = *R*, *P*_FA_ = 0 for very high values of *c* (i.e., where not even the very strongest feelings of familiarity are sufficient to trigger investigation; only recollection can). As can be seen in the equations, the Recollection parameter specifies the intercept of the ROC curve with the *P*_hit_ axis. In other words, it defines the proportion of hits that would be achieved even if no sense of Familiarity is strong enough to trigger retrieval. If d’ = 0, the ROC curve is a straight line between the intercept of the *P*_hit_ axis and the top right hand corner; if d’ > 0, it arcs upwards (cf. ROC curves in Fig. 1). We note that points with high decision criteria are therefore more informative about the value of *R*.

Of course in reality, we cannot re-run experiments, keeping each observer’s memory performance the same while varying their decision criterion. But self-reported confidence enables us to *simulate* this. Our human observers only investigated locations (opened canisters) for which the sense of familiarity exceeded some baseline decision criterion, or which they recollected. The resultant hit and false alarm rate gives us our first point on the ROC curve. If we now pretend that they did not investigate locations for which they expressed low confidence, we obtain a new pair of *P*_hit_, *P*_FA_, representing a second point on the same ROC curve but with a higher decision criterion (thus, shifted left/downwards). If we do the same retaining only decisions made with high confidence, we obtain a third point with still higher decision criterion. To obtain estimates of *d’* and *R*, we fit the ROC equations to these 3 data points using the Maximum Likelihood method. Fitted *d’* was constrained to be ≥ 0, since negative *d’* would mean performance systematically worse than chance. The values of *d’* and *R* together specify the area under the ROC curve (AUROC), which is a measure of overall performance independent of memory type. If there is no memory at all (*d*’ = *R* = 0), the area is 0.5. If memory is perfect (either *R* = 1, or *d*’ >  > 1), the area is 1.

This way of assessing memory using self-reported confidence (Confidence method; see Supplementary Materials) is well established, but was only available for our human participants. For both humans and birds, we also used the order in which they searched locations as a proxy for confidence (Order method; see Supplementary Materials). We assumed that the first third represented the most confident decisions, corresponding to the highest criterion, with the second and final thirds representing medium and low confidence/criteria, respectively. In this way, we again obtained 3 pairs of *P*_hit_, *P*_FA_, to which *R*, *d*’ could be fit. In humans, we then compared the memory parameters estimated with the gold-standard Confidence method with our novel Order method. This validated the Order method as a way of measuring memory performance in non-verbal participants. This is important since for the birds, only the Order method was available.

### Definition of Recollection and Familiarity memory components, and of memory enhancement

These three metrics (*d*’, *R*, AUROC) are poorly suited for comparing the respective contributions of familiarity and recollection to overall memory performance, since they all span a different range. *d’* runs from 0 (chance, since we did not allow fits below chance) with no upper bound; *R* runs from 0 (no recollection) to 1 (perfect recollection); AUROC runs from 0.5 (chance, since we did not allow fits below chance) to 1 (perfect memory). Thus, we first converted them to a common range [0,1], in a process shown graphically in Fig. 1. In place of AUROC, we define Overall Performance = 2*AUROC-1 (pink region in Fig. 1D). To compare a given pair of values *R* and *d’*, we considered 2*AUROC_R_-1 and 2*AUROC_d_-1, where AUROC_R_ is the area under an AUROC with the given value of *R* but *d’* = 0 (Fig. 1B), and AUROC_d_ is the area with the given *d’* but *R* = 0 (Fig. 1C). It is easy to show that 2*AUROC_R_-1 is in fact just *R*, which we call Recollection (red region in Fig. 1E). 2*AUROC_d_-1 is our measure of Familiarity (green region in Fig. 1F). Where Overall Performance is less than around 0.75, Overall Performance is approximately equal to the sum of Recollection + Familiarity (and where performance is high, it is impossible to discriminate Recollection and Familiarity anyway).

Familiarity might be positive even in the Naïve condition, if subjects consider some hiding places intrinsically more plausible than others. Furthermore, since we did not allow fitted ROCs to drop below chance, the mean of the Naïve parameters must necessarily be positive. Thus, to assess memory it is important to look at how performance is enhanced in the Retrieval condition compared to the Naïve condition. Finally, therefore, we then subtracted the Naïve value from the Retrieval value to give us Overall Enhancement ((2*AUROC-1)_Ret_—(2*AUROC-1)_Naïve_; pink region in Fig. 1G), the Familiarity Enhancement ((2*AUROC_d’_-1)_Ret_—(2*AUROC_d’_-1)_Naïve_; red region in Fig. 1H) and Recollection Enhancement ((2*AUROC_R_-1)_Ret_—(2*AUROC_R_-1)_Naïve_; green region in Fig. 1I).

### Statistical analysis

Having expressed Familiarity and Recollection Enhancement on a common scale, we then compared the contributions of these two types of memory in both humans and birds. For this comparison, we used only the 1-day retention interval for the birds, the same as for the human data. We fitted a Linear Mixed Model, Enhancement ~ Metric * Group + (1|DistribID), where Metric was a categorical within-subject factor specifying the metric under consideration (Familiarity vs. Recollection), and Group was a categorical between-subject factor (birds at the 1-day retention interval vs Humans in the Choice condition vs. Humans in the Random condition), and DistribID was a random factor corresponding to the distribution of seeds/coins. We used function lmer of R package lmerTest; code is provided in StatsAndFigures_CompareHumansAndBirds.R. Estimated marginal means were obtained using function emmeans of package emmeans; pairwise comparisons were done without adjustment for multiple testing.

To examine only the bird data, we again fitted a Linear Mixed Model, Enhancement ~ Metric * logdelay + (1|DistribID), with Metric again being a categorical within-subject factor (Familiarity vs. Recollection), and the log_10_ of the retention interval as a co-variate. Code is provided in StatsAndFigures_Birds.R. Any other tests used are standard and are mentioned in the Results section. Results were considered significant if p < 0.05.

## Results

### Validation in human participants

**Fig. 2:**
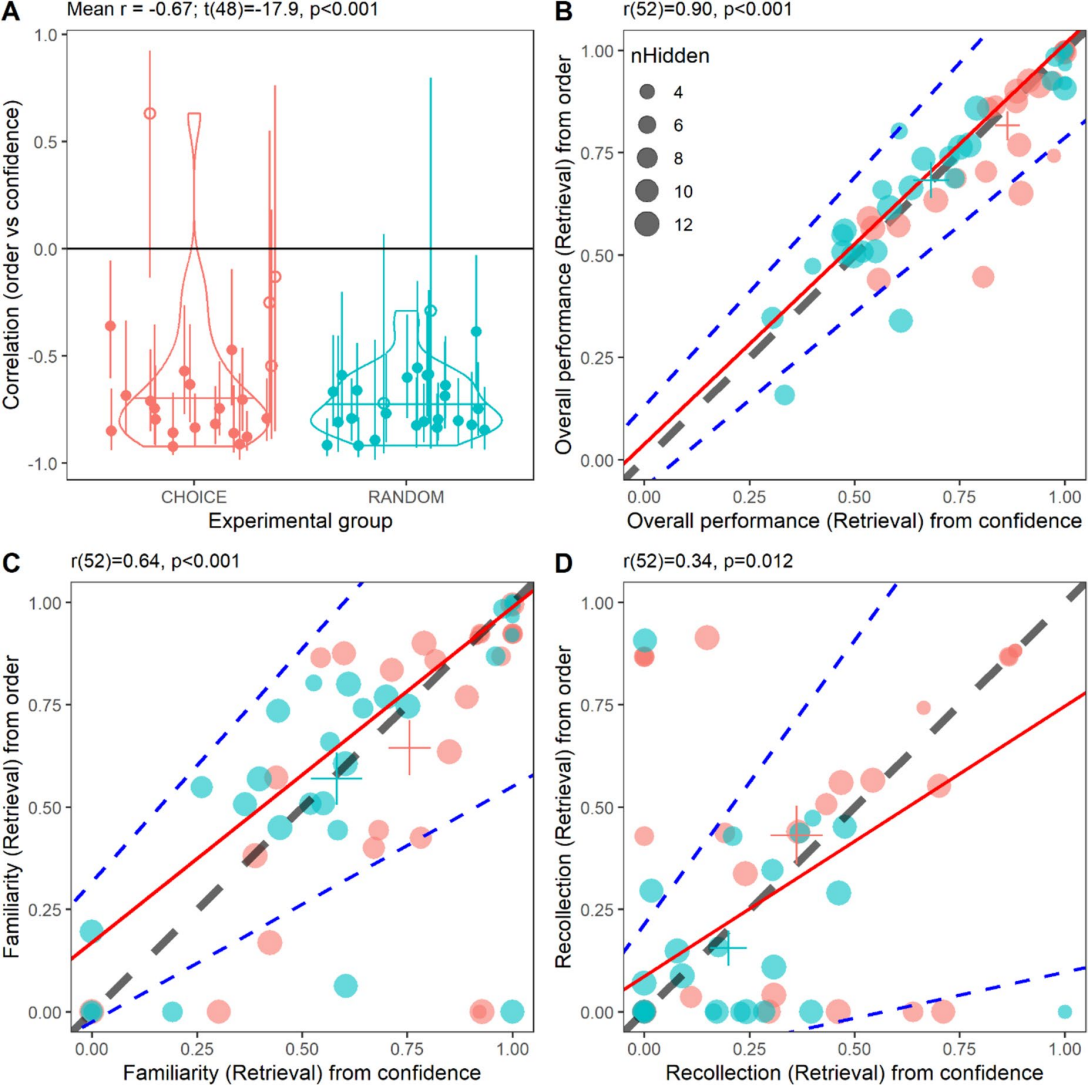
Outcomes of human validation trials, comparing Confidence and Order metrics for the Retrieval condition. **A**: Distribution of participants’ individual Pearson correlation coefficients between the order in which they selected targets and their reported confidence, for the 49/54 participants who used more than one confidence rating. In obtaining these correlations, all searches were used (i.e., we did not truncate if the number of false alarms exceeded the number of objects hidden, unlike what we did for fitting the ROC curves). Error bars show the 95% confidence interval on individual correlation coefficients. 43/49 individual correlation coefficients were significantly negative (p < 0.05, filled symbols). Overall, the whole population was very significantly negative t_48_ = -17.9, p < 0.001. Results for the Choice and Random group are plotted separately but there was no difference between them (t_29_ = -1.09, p = 0.29, two-sample t-test; colored horizontal lines show the median for each group and the outline represents the data distribution). **B**–**D** Scatterplot of Overall performance, Familiarity, and Recollection derived from Order scores of human participants against Overall performance, Familiarity, and Recollection derived from reported Confidence (gray dashed lines are the identity lines; continuous red lines are linear fits using Type II regression, blue dashed lines show the lines resulting from the 97.5% intercept and slope, and the 2.5% intercept and slope; large crosses show group mean ± 1SEM; pink, blue are for participants in the Choice, Random groups, respectively). Symbol area represents number of coins hidden. Titles report Pearson correlation coefficients and significance. [This figure was generated by file StatsAndFigures_HumanValidation.R] (color figure online)

We first tested our assumption that the order in which decisions were made can be used as a proxy for confidence levels. Human participants made earlier decisions with higher confidence than later decisions (individual correlations between confidence (scored 3 for “sure”, 2 for “probably” and 1 for “guess”) and order were significantly negative in 43/49 participants; mean correlation across participants: r = -0.67, *t*(48) = -17.9, *p* < 10^–10^: Fig. 2A; correlation could not be computed in an additional 5 participants as they only used one confidence level). This supports the idea of using order as a proxy for confidence. All memory measures correlated significantly between the two methods of producing ROC curves (gold-standard “Confidence” method and novel “Order” method), and the estimates of the two methods were not significantly different from each other: Overall performance (Pearson correlation *r*(52) = 0.90, *p* < 10^–10^; Wilcoxon signed rank test *p* = 0.26, Fig. 2B), Familiarity (*r*(52) = 0.64, *p* < 10^–6^; Wilcoxon signed rank test *p* = 0.32, Fig. 2C), and Recollection (*r*(54) = 0.34, *p* = 0.012; Wilcoxon signed rank test *p* = 0.96; Fig. 2D). Thus, the Order method yields substantially the same results as the “gold standard” method using self-reported confidence; there is some variability, but no evidence of systematic bias. For further analysis of the noise and (non-existent) bias in our method, see Supplementary Materials.

### Comparison of humans and birds

**Fig. 3:**
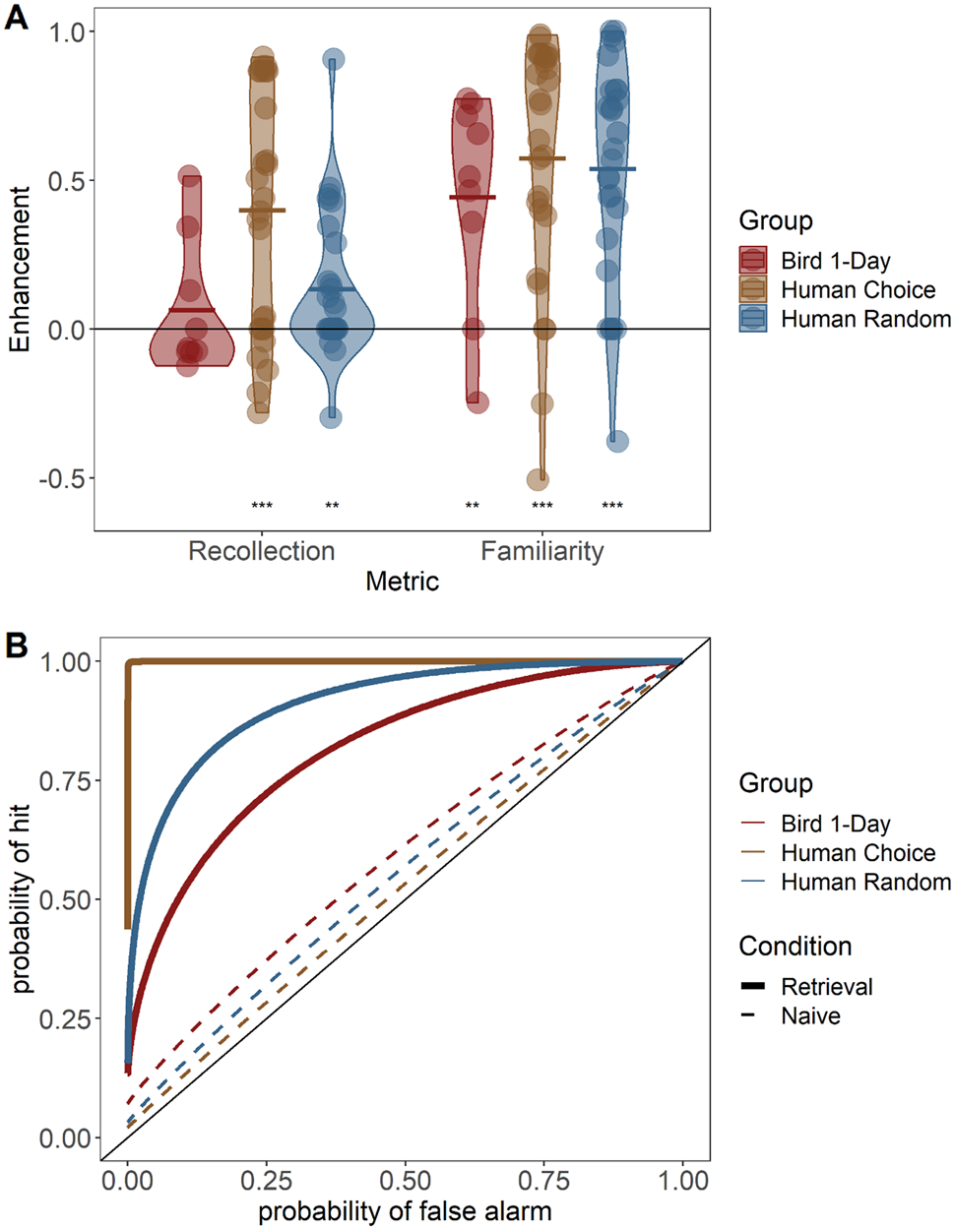
**A** – Distributions of Recollection and Familiarity Enhancement, for birds in the 1-day retention interval and for human participants in Choice and Random groups. Horizontal bars show the mean of each distribution. The horizontal line through 0 represents same performance during the Retrieval and Naïve conditions, i.e., no memory. Asterisks indicate whether the values were significantly different from zero (t-test; ** p < 0.01, *** p < 0.001). **B** – Average ROC curves, using the mean d’ and R for each group/condition. [This figure was generated with StatsAndFigures_CompareHumansAndBirds.R]

Since we cannot use the gold-standard Confidence method (using explicit confidence ratings) for the birds, we compared the humans’ performance to the birds’ performance at the 1-day retention interval using only the Order method (Fig. 3). As described in the methods, the fit parameters *d’* and *R* for both the Retrieval and Naïve conditions were used to obtain the Familiarity Enhancement and Recollection Enhancement due to memory. Comparing these to each other, humans in the Choice condition outperformed both humans in the Random condition, and birds, whereas humans in the Random condition did not perform significantly differently from the birds (F_2,60_ = 3.90, p = 0.0256; Pairwise comparisons: Choice vs. bird: p = 0.02; Choice vs. Random: p = 0.034; Random vs. bird: p = 0.398). In all cases, Familiarity Enhancement was significantly higher than Recollection Enhancement (F_1,60_ = 19.47, p < 0.001). The interaction between group and memory index was not significant (F_2,60_ = 1.53, p = 0.2253). Looking at individual groups and metrics, both Familiarity and Recollection Enhancements were significantly above zero for both human groups (Familiarity: Random t_26_ = 7.55, Choice t_26_ = 7.22, both p < 0.001; Recollection: Random t_26_ = 2.88, p = 0.008; Choice t_26_ = 5.08, p < 0.001; two-tailed t-test). In the birds, Familiarity Enhancement was also significantly above zero (t_8_ = 3.74, p = 0.006) but Recollection Enhancement was not (t_8_ = 0.85, p = 0.42).

### Coal tit cache memory at different retention intervals

**Fig. 4:**
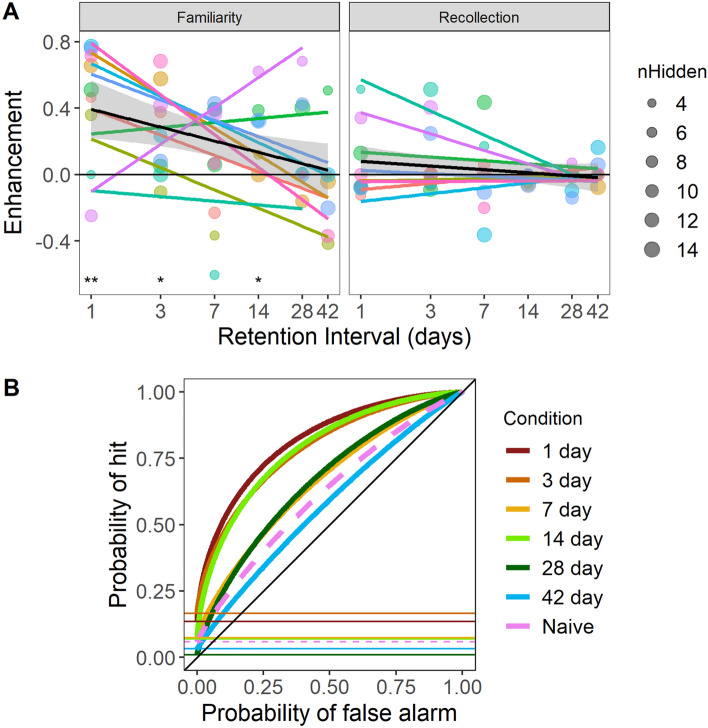
**A**. Memory Enhancement for coal tits as a function of retention interval. Colors identify the 9 different Retrieval birds. The size of the symbol represents the number of seeds hidden (nHidden), which was out of our control and determined by the birds’ behavior. Colored lines show the fitted regression for each Retrieval bird; the black line + ribbon shows the regression fitted to all birds, ± SEM. Asterisks above the axis labels indicate whether the Enhancement was significantly different from zero for the given retention interval (* p < 0.05, ** p < 0.01). **B**: Average ROC curves for bird groups. The thick dashed pink curve shows the ROC curve computed with the mean R and d’ averaged from all Naïve birds in all “delays” (i.e., seed distributions). The thick colored lines show ROC curves computed with the mean R and d’ averaged over all available Retrieval birds at the specified retention interval, i.e., 1, 3, 7, 14, 28, and 42 days after caching, as indicated by the colors. The horizontal lines mark the y-intercept in each case, corresponding to the Recollection parameter R. [This figure was generated by file StatsAndFigures_Birds.R] (color figure online)

Analyzing only the bird data, for all retention intervals, Familiarity Enhancement was significantly higher than Recollection Enhancement (F_1,82_ = 10.67, p = 0.0018). Investigating the parameter estimates shows that the Recollection Enhancement was no different from chance (p = 0.2558). Performance declined significantly with retention interval (F_1,84_ = 9.47, p = 0.0028). Although Recollection Enhancement did not significantly decline with retention interval (p = 0.3564), this decline also did not differ significantly from the decline of the Familiarity Enhancement (Interaction: F_1,82_ = 3.18, p = 0.0782; Fig. 4A). A few birds seem to have produced higher Recollection Enhancements for the shorter retention intervals (Fig. 4A), but it is unclear whether this is due to individual differences or “noise” in the ROC curve fitting. A simulation power analysis (see Supplementary Materials) found that with the small number of decisions and the small number of birds available to us to reconstruct the ROC curves, we should have been able to detect a Recollection Enhancement of 0.3 or above. The overall memory enhancement detected at the low retention intervals, if all had been due to Recollection instead of Familiarity, would have resulted in a Recollection Enhancement of 0.52, which would be well within our ability to detect.

## Discussion

We have shown that when humans hide objects in a large lecture theater, their memory for locations is mostly based on Familiarity, with a smaller contribution of Recollection. In food-hoarding birds as well, contrary to our predictions, Familiarity was by far the stronger contributor to spatial memory compared to Recollection. In fact, we were not able to detect a significant contribution of Recollection in the birds. However, birds also did not differ significantly from the humans in the Random group in their performance. This means we should be cautious in our interpretation, as the perceived lack of Recollection in the birds could be a question of statistical power due to the smaller sample size. The fact remains, however, that the contribution of Familiarity was significantly higher than that of Recollection, in both birds and humans.

This is the first study to use ROC analysis of Familiarity and Recollection on spatial memory in any species. With spatial memory, all locations have to be presented at the same time, as they are all part of the spatial context for the other locations. Simultaneous presentation makes it possible to let the participants decide for themselves in which order to search the locations. We show that the order in which humans make these decisions can be used as a proxy for their confidence levels, and can therefore be used to construct ROC curves. While we do not have proof that this correlation between order and confidence also exists in coal tits, as explained in the Introduction, this is not an unreasonable assumption. Hoarding birds are known to make fewer mistakes when retrieving their first items compared to later items, suggesting they go for the items that are better remembered first (Kamil and Balda 1990). Although the assumption is not unreasonable, it has to be kept in mind that this is an important assumption, and that other factors may influence the order in which animals (and humans) may explore an array of locations or stimuli. If some stimuli are easier to investigate than other (e.g., lower cost, closer by), then they might be investigated first. Similarly, if all items are very close together, the cost of making “false alarm” mistakes may be so low that the order in which locations or stimuli are investigated does not reflect confidence anymore. Indeed, in previous version of the task in humans (data not shown), when locations were close together, and the people barely had to move to investigate them (e.g., 20–30cm apart on a wall), people had a tendency to go through the stimuli systematically, always moving to a neighboring location. We overcame this confound by (1) marking the locations that had already been searched (part of the motivation for the systematic search is to not go back to the same item over and over again, so the participants told us), by (2) using a space that is large, relative to the person, so they have to actively move from location to location, and (3) by only allowing a limited number of searches, such that the cost of making mistakes was higher. In the birds as well, these conditions were fulfilled: (1) they pulled the strings aside, marking the site as searched, (2) they had to fly from perch to perch, and (3) they only had 4 min to search for the caches. This approach opens up many new opportunities to study the use of Familiarity vs. Recollection in populations that cannot provide explicit confidence scores, both human (e.g., small children) and non-human (as in this study). The stimuli do not need to be spatial locations. The same approach can be used with other simultaneously presented stimuli (words, pictures, objects), although one would need to be sure to present them in different locations in the retrieval phase than in the memorization phase, to prevent the confound of spatial memory. The conditions would also need to be right to assure the correlation between order and confidence.

In our human experiment, we had two different sets of instructions: people either got to choose where they hid their coins, or they were told exactly where to hide them. We did this because we could imagine that people would try to simplify the task using rule-based strategies that would be simpler to remember, yet still difficult to search for other individuals. Although we do not know what these rule-based strategies were for the different participants (we did not ask them), there is some evidence in our data that they may indeed have done this. The Choice group had better overall memory performance than the Random group, and this was the case both in terms of their Recollection and their Familiarity. Although the interaction was not significant, the figure indicates that the effect was most prominent in the contribution of Recollection enhancement. Importantly, both groups showed significantly above chance Recollection and Familiarity performance, showing that humans use a combination of both memory systems to solve spatial memory problems.

We did not find support for our hypothesis that food-hoarding birds would use Recollection as the primary memory mechanism for cache retrieval. In fact, we found the opposite, with no clear evidence for the consistent use of Recollection (with the caveat that our sample size was small; see simulations in the Supplementary Materials), but good evidence for the use of Familiarity. We also found that memory performance based on Familiarity declined over time. Performance based on Recollection, being indistinguishable from chance performance, did not decline significantly over time, although the decline was also not significantly different from that of Familiarity, probably again due to our low statistical power. It is therefore possible that birds do use Recollection to some degree, and that it also declines over time. Nevertheless, the contribution of Familiarity is significantly higher.

The coal tit result was similar to that in adult humans: after a 1-day retention interval, Familiarity is the dominant contributor to the spatial memory. The role of Recollection is significantly smaller than the role of Familiarity. It would be interesting to see if Recollection would have a stronger contribution for shorter retention intervals (i.e., within the same day), as previous work using Remember/Know paradigms has shown that the number of “Remember” responses drops significantly in the first 24 h, while the performance based on “Know” (i.e., Familiarity) declines much more gradually, possibly without change for up to 2 weeks (Gardiner and Java 1991; Tunney 2010; Tunney and Bezzina 2007). In our coal tit data as well, Familiarity performance at 14 days was similar to performance at 3 days and declined after that. The lack of evidence for Recollection could possibly be due to a very rapid Recollection decline in the first 24 h after caching. However, if that is the case, this memory system would not contribute significantly to the longer-term retrieval of caches in the field.

Our findings that coal tits use mainly Familiarity could theoretically be an artifact of the captive situation in which we tested the birds’ memory. The limited space which was reused for all 6 trials and the similarity of cache sites may have made Recollection more difficult. However, theorists have argued that Recollection is much less sensitive to interference than Familiarity (Sadeh, Ozubko, Winocur, and Moscovitch 2016), making this unlikely. Given the ecology of wintering titmice and chickadees, Familiarity may indeed be a better mechanism to use than Recollection when retrieving caches. First, most food-hoarding Parids defend a winter flock territory, and move slowly around that territory while foraging (Ekman 1989). That means that not only do they cache all through the flock territory, but at any point in time, they are also always foraging near a location where they have cached food previously. It is, therefore, highly likely that they will come across a recent cache location as part of their natural foraging effort. Indeed, crested tits (*Lophophanes cristatus*) have been shown to cache food in locations where they individually are most likely to forage later in winter (Lens, Adriaensen, and Dhondt 1994). Second, because the birds move around in a flock, it might be difficult for any given bird to act on a Recollection event if the recollected site is away from the flock, as individuals never venture far from the flock for safety reasons. Finally, the Familiarity mechanism is thought to involve less cognitive effort than Recollection (Basile and Hampton 2013; Jacoby 1991), and therefore may be more efficient in terms of time spent retrieving, as Familiarity will automatically flag up cache sites when the bird comes across them in the process of normal foraging.

Of course, Familiarity cannot account for the totality of cache memory. Sites from which they had recently removed caches would be more familiar (this being their second visit) and would therefore be visited more often if the sense of Familiarity was all they used. In reality, the birds know which sites they have already emptied and which they have not (Sherry 1984a), as well as what kind of food was hidden in that cache and when it was placed there (Feeney et al. 2009, 2011). Familiar spatial context is well known to elicit explicit memories of events that occurred in that context (Robin, Garzon, and Moscovitch 2019). We, therefore, speculate that the memory for the spatial locations themselves is largely based on Familiarity, but that the familiar location then triggers an episodic-like memory about the content of the cache, and potentially even when the cache had been placed there (Feeney et al. 2009).

The importance of the hippocampus in Recollection has been demonstrated in previous studies investigating the dual process model such as in amnesic humans with hippocampal damage (Yonelinas et al. 1998). In a study with rats, animals that underwent hippocampal lesions showed reduced Recollection for olfactory memories: the generated ROC curves became symmetrical, displaying a reliance on Familiarity only in these rats (Fortin et al. 2004). While these rat studies suggest that Recollection (of olfactory information) requires the hippocampus whereas Familiarity does not, other work suggests that in humans, the hippocampus may play some role in Familiarity as well as Recollection. In a fMRI study, activation was found in the hippocampus of human participants when utilizing Familiarity in a memory test (Wais, Squire, and Wixted 2010). Similarly, it has been proposed that the hippocampus is utilized in Familiarity when the familiar memory is as strong as a Recollection memory (Smith, Wixted, and Squire 2011). Finally, it has been argued that the hippocampus has an important role in the processing of spatial information, regardless of the memory mechanism involved (Hartley, Lever, Burgess, and O'Keefe 2014; Moser, Moser, and McNaughton 2017), and no studies, to our knowledge, have investigated the role of the hippocampus in the Familiarity processes with regards to spatial memory. But even if the hippocampus is not involved in Familiarity at all, if the familiar spatial location triggers an episodic-like memory (or at least a memory of the content of that cache), this process itself may rely on the hippocampus (Robin et al. 2019). This suggests that our finding is not inconsistent with the fact that lesion of the hippocampus in food-hoarding birds prevented successful cache retrieval (Sherry and Vaccarino 1989), and that food-hoarding birds have greater hippocampal volume than non-hoarding species (Krebs et al. 1989; Sherry et al. 1989). The latter could relate to the number of episodic-like memories they need to retain, associated with familiar locations.

Parids are of course not the only food-hoarding birds. Corvids have been studied extensively for their memory for cache sites, content, and temporal context. We expect that Recollection may play a more important role in cache relocation in Corvids. Unlike most Parid species, most hoarding Corvids do not forage in cohesive flocks through a winter territory. In those circumstances, the advantage of using Familiarity may be reduced, and it may indeed be more advantageous to use Recollection to remember where a particular food item has been hidden and then to go and access it. This would be especially the case for solitary caching birds, such as Clark’s nutcrackers (*Nucifraga columbiana*) which can place caches up to 32km from their home range (Lorenz, Sullivan, Bakian, and Aubry 2011). When relocating caches over long distances, Recollection would be expected to be more important, so that the bird does not waste energy traveling these distances in search of a familiar location. Even then, it is still possible that the localization of the precise cache site uses Familiarity mechanisms once the birds are in the correct area. Similarly, for more locally caching Corvids like magpies (*Pica pica*) or scrub jays (*Aphelocoma californica*), the question of whether they use Familiarity or Recollection to relocate cache locations remains open; linking those locations to content and a caching episode (Clayton, Yu, and Dickinson 2001), however, is very likely to use Recollection.

## Conclusions

We conclude that food-hoarding Parids mainly use a Familiarity process to remember the locations of cache sites, although the Recollection mechanism could contribute to spatial memory as well, be it less importantly. We hypothesize that episodic-like Recollection processes may be involved in linking the identified spatial locations with their content, and that this may be the main function of the enlarged hippocampus. This hypothesis remains to be tested, however.

### Supplementary Information

Below is the link to the electronic supplementary material.

Supplementary file1 (DOCX 1174 KB)

## Data Availability

The data presented in this paper, as well as all the individual ROC curves, can be found here: https://doi.org/10.25405/data.ncl.14938098.v1. The analysis code, including the code for ROC fitting can be found here: https://doi.org/10.25405/data.ncl.22710376.v1.
