# Supplementary material for: Hoarding titmice predominantly use Familiarity, and not Recollection, when remembering cache locations
Source: Anim Cogn. 2023 Oct 21;26(6):1929–43. doi: 10.1007/s10071-023-01829-3 (PMC10769918; doi:10.1007/s10071-023-01829-3)
Supplement: Supplementary file 1 — Supplementary file1 (DOCX 1174 KB) [file 10071_2023_1829_MOESM1_ESM.docx]

**SUPPLEMENTARY MATERIALS**

**Hoarding titmice predominantly use Familiarity, and not Recollection, when remembering cache locations**

Tom V. Smulders, Laura J. Douglas, Daniel Reza, Lucinda H. Male, Alexander Prysce, Amélie Alix, Alexander de Guzman Dodd, and Jenny C. A. Read

SUPPLEMENTARY METHODS

1. Layout of the study environment for the human study


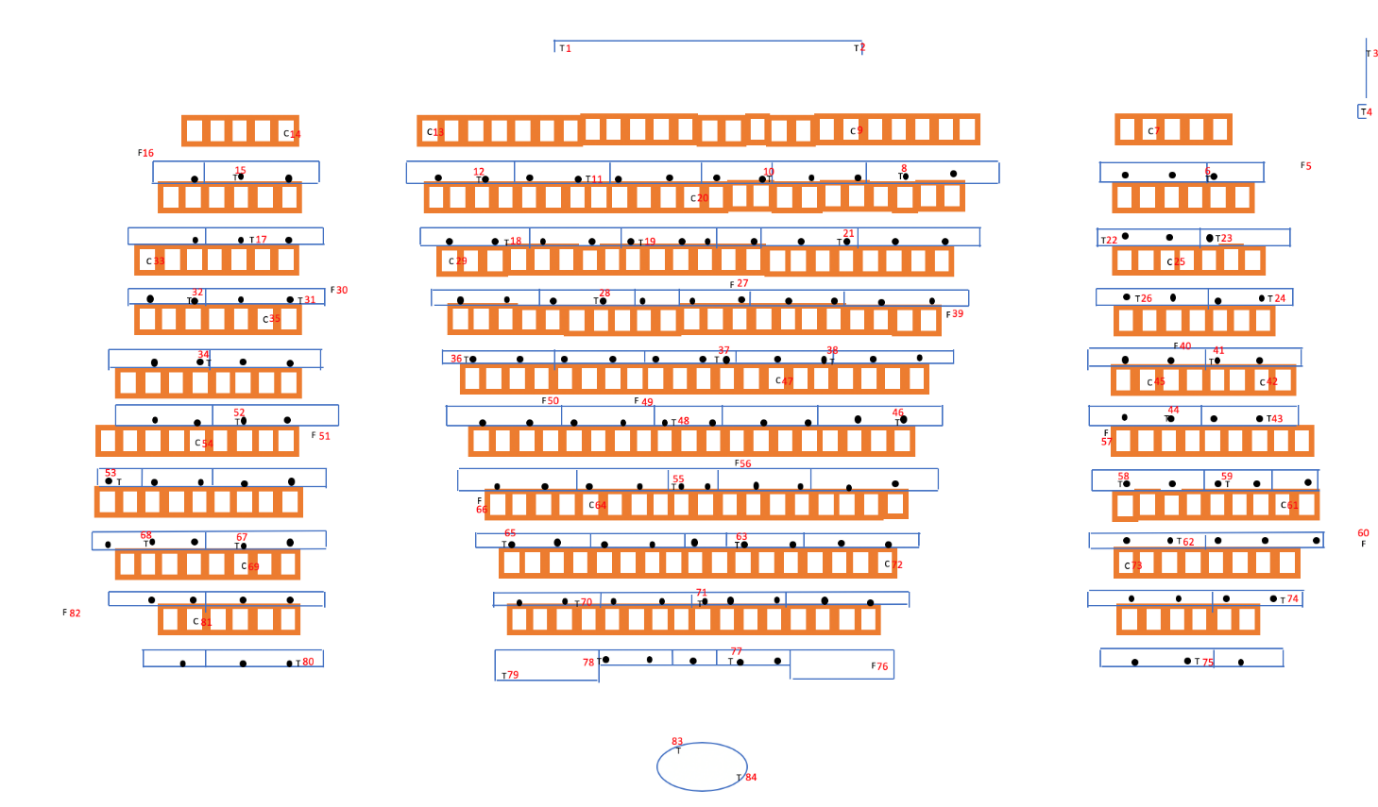


***Figure S1. Layout of the room used for the human validation study. Orange squares represent seats, blue rectangles desks, black disks power ports, and red numbers the locations of the film canisters (T=table; C=chair; F=floor).***

2. Maximum likelihood fitting

Suppose we present a given number of targets, of which *N*­_hit_ are successfully detected and *N*_miss_ are missed. The probability of observing these results is $\left( \begin{matrix} N_{hit}+N_{miss} \\ N_{hit} \end{matrix} \right)P_{hit}^{N_{hit}}P_{miss}^{N_{miss}}$, where the first term is the binomial coefficient, P_hit_ is given in Equation 1 in the main paper and *P*_miss_=1-*P*_hit_. *P*_hit_­ and *P*_miss_ depend on the observer’s own peculiar *R* and *d*’, and also on the decision criterion they adopted on this particular occasion, which we will call *c*_j_; thus $P_{Hit}=P_{Hit}(R,d^{'},c_{j})$.

We can write down a similar term for the probability of getting *N*_FA_ false alarms and *N*_CR_ correct rejects out of the given number of lures. The likelihood of observing N­_hit_­ hits and N_­FA_ false alarms is the product of these two probabilities. The log-likelihood of this set of observations is

${\log L}_{j}(R,d^{'},c_{j})=N_{hit}P_{hit}+N_{miss}P_{miss}+N_{FA}P_{FA}+N_{CR}P_{CR}+K$

where the term *K* represents the total log binomial coefficients and the subscript j reminds us that this set of observations is assumed to be for decision criterion c_j_. Now suppose we have *M* sets of (*N*_hit_,*N*_miss_,*N*_FA_,*N*_CR_), representing different points on the ROC curve and thus different values of the decision criterion (Fig. S2A). The log-likelihood for the whole set of *M* points is thus

$$\log L_{ROC}=\sum_{j=1}^{M} \log L_{j}\left( R,d^{'},c_{j} \right)$$

We fit the data by searching for the values of *R*, *d*’ and *c_j_* which maximise this log-likelihood, subject to the constraints 0≤*R*≤1 and *d*’≥0. We do this in MATLAB using the function fminsearch to minimise the negative log likelihood, with the constraints imposed by setting this to a large value when the constraints are violated, and MaxFunEvals set to 5000. We use fminsearch to optimise the two parameters *R* and *d*’. Within each function evaluation for a particular pair of *R* and *d*’, we call fminsearch again to optimise the *M* parameters *c_j_*_­_ for each set of (*N*_hit_,*N*_miss_,*N*_FA_,*N*_CR_) (see example in Fig. S2B).

In optimisation problems, the initial guess is often critical. Here, we obtain an initial guess for *R* and *d*’ by averaging (*N*_hit_,*N*_miss_,*N*_FA_,*N*_CR_) across all *M* points and assuming this represents the point on the ROC curve where *c=d’*/2, i.e. the decision criterion is exactly halfway in between the mean Familiarity for targets and lures. Using Equation 1 and Equation 2 with this value of *c*, we can then estimate initial guesses as

$$\hat{P}_{Hit}=\frac{<N_{hit}>}{<N_{hit}+N_{miss}>}=\hat{R}+(1-\hat{R})\Phi\left( +\frac{\hat{d}'}{2} \right)$$

$$\hat{P}_{FA}=\frac{<N_{FA}>}{<N_{FA}+N_{CR}>}=\Phi\left( -\frac{\hat{d}'}{2} \right)$$

These equations can be solved for $\hat{R}$ and $\hat{d}'$, which become the initial guesses provided to fminsearch (except that if these equations yield negative values of either parameter, we set the initial guess to 0). We then run fminsearch a second time, this time with the initial guesses being the values of *R* and *d*’ output by the first run.

With only a few points on the ROC curve, each of which being obtained with low *N* and thus having large error bars (Fig. S2A), there are considerable uncertainties on the fitted parameters *R* and *d*’. We estimated the 95% confidence interval for each parameter by looking at the range of values for which the log-likelihood is within 1.92 of its maximum value (likelihood ratio test using Wilke’s theorem). This is shown by the green area in Fig. S2C. To find this area, we fix *R* to one of several values in turn and optimise *d*’ only. If the log-likelihood at this point is within 1.92 of the maximum, we mark the point with a purple dot in Fig. S2C, and then move *d’* above and below this value until either the log-likelihood falls below 1.92 of maximum, or *d*’ falls below 0. This gives us the vertical extent of the confidence region at the chosen value of *R*. Finally, we draw a box around the confidence region and take its edges as being the 95% confidence intervals on fitted *R* and *d*’ (red error bars in Fig. S2C). The additional ROC curves drawn on Fig. S2A give an idea of the range of fits within this confidence region.


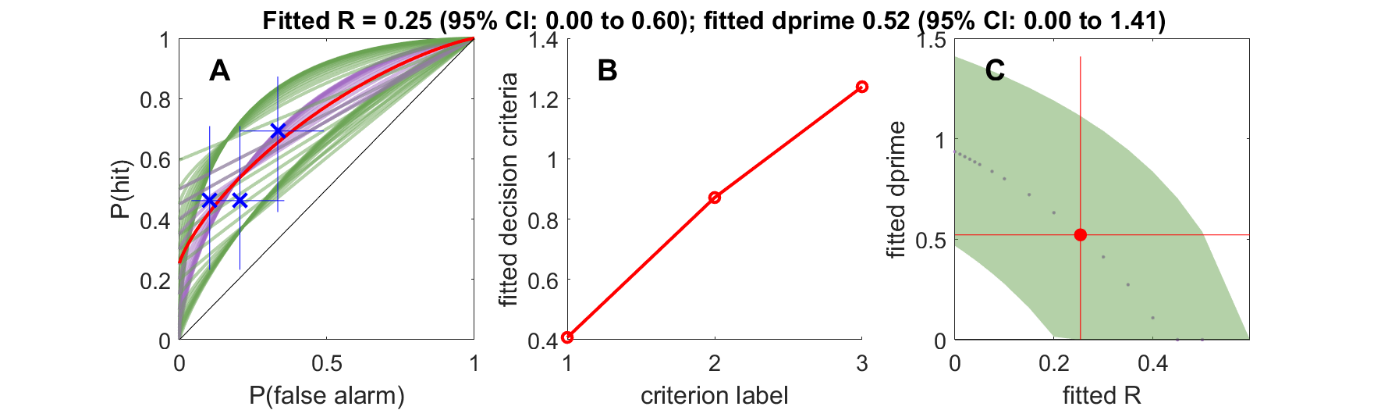


***Figure S2. Fit for human subject 2. (A) Blue crosses: M=3 estimated points on the ROC curve, along with 95% confidence intervals obtained using the Score algorithm for binomial confidence intervals. Heavy red line: ROC curve for the R and d’ parameters that maximise the likelihood of obtaining the data shown with blue crosses: R=0.25, d’=0.52. Green lines show example ROC curves corresponding to the upper and lower edge of the confidence region in C; purple lines show ROC curves corresponding to the optimal d’ when R is constrained to different values within the confidence region (purple dots in C). (B) Values of the fitted decision criteria c_j_ for each of the three points. (C) Large red dot shows the maximum-likelihood R and d’. Small purple dots show the d’ that maximises the likelihood when R is constrained to the value indicated on the horizontal axis. Green region shows the estimated confidence region, within which the log-likelihood remains within 1.92 of its maximum value. Red error bars mark the maximum and minimum extent of this region along the individual axes.***

3. Fitting human data using self-reported confidence

Human participants gave confidence judgements (sure, probable, guess) for each stimulus they thought was a target. We used these to infer performance for different decision criteria and thus estimate different points on the ROC curve (Fig. S3). The first point (right-most on the ROC curve) uses their actual judgments, taking “hits” to be all targets which they correctly selected as such, and “false alarms” to be lures they wrongly selected, regardless of confidence. The second point (to the left of the first point) simulates a higher decision criterion, labelled C_prob_ in Fig. S3, by now classing only stimuli selected as “probable” or “sure” targets to be hits or false alarms; stimuli selected as “guess” targets were now classified as not selected. The third and left-most point, similarly, simulates a still higher criterion by including only stimuli classed as “sure” targets, with the other stimuli classified as not selected. In this way, we obtain 3 sets of (*N*_hit_,*N*_miss_,*N*_FA_,*N*_CR_) and thus 3 points on each subject’s ROC curve (Fig. S2A). As noted above, increasing the decision criterion reduces *P*_Hit_ and/or *P*_FA_ , and so corresponds to moving downward/leftward along the ROC curve.


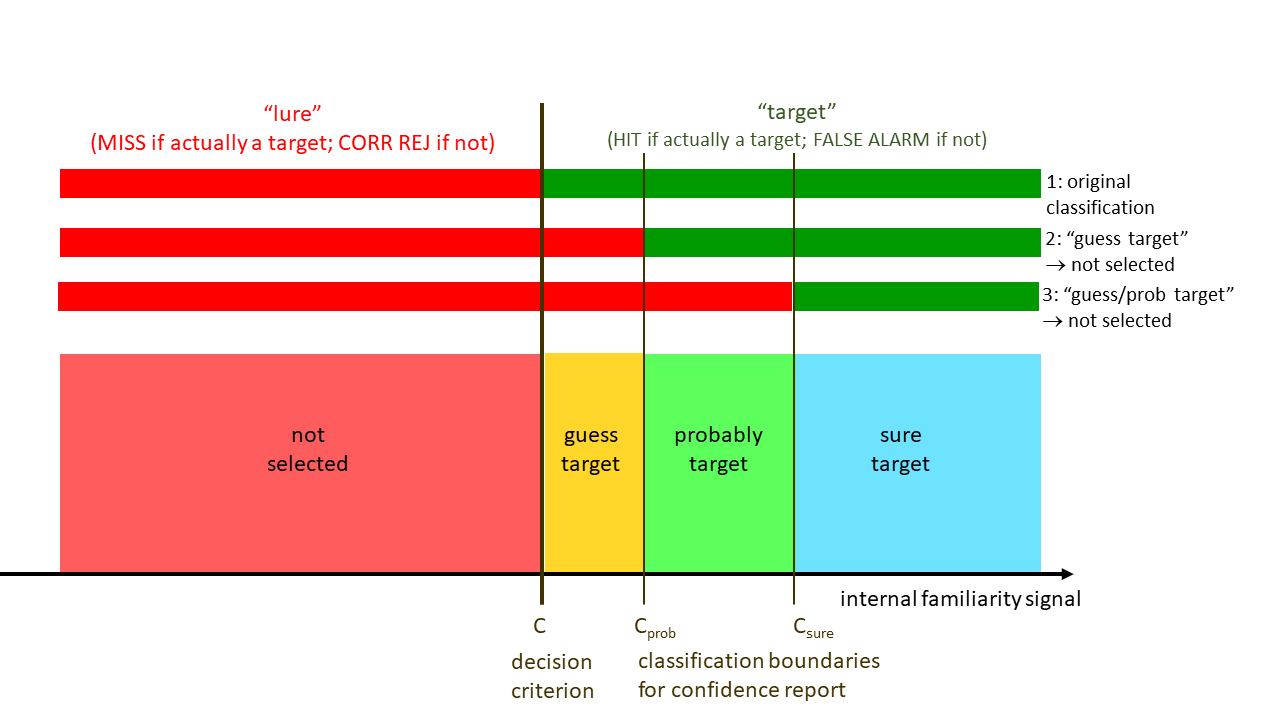


***Figure S3. Illustration of how we used confidence judgments to generate new points on the ROC curve. We assume that for targets that are not recollected, participants use an internal Familiarity signal. If this signal is below the decision criterion C, they do not select the item; this judgment is therefore correct if the item was a lure but a miss if it was a target. If the signal is above C, they select the item as a target. If the signal is in between C and C_prob_ (yellow region), the reported confidence is “guess”; if it is between C_prob_ and C_sure_ (green region), the confidence is “probably”; if the signal is above C_sure_ (blue region), the confidence is “sure“. As shown across the top, the first point on the ROC curve is obtained by using the original binary classification. The second and third points are obtained by reclassifying first “guess target”, and then both “guess/probably target” responses as “lure” responses.***

4. Fitting human data using order of decision

Here, we only use the stimuli which humans selected as targets. These were therefore either “hits” (actually targets) or “false alarms” (actually lures). As noted above, we ignored any selections beyond the N_tgt_^th^ false alarm, effectively classifying them as correct rejects or misses rather than as false alarms or hits. As an example, for one subject, this process results in 23 stimuli selected in the recall phase: 10 hits and 13 false alarms, in the sequence HFHFFHHHHFHHH|FHFFF|FFFFF (see below for the significance of the bars |). The first point on the ROC curve is obtained by simply using all these: *N*_hit_=10, *N*_FA_=13 (and thus *P*_hit_=0.77, *P*_FA_=0.33). To simulate raising the decision criterion, we now “deselect” stimuli from the bottom up (since these are assumed to be those where the Familiarity signal was lowest, which would be removed by a higher decision criterion; Fig. S3). We do this in two phases, each designed to remove roughly a third of the false alarms. Since in this example, *N*_FA_=13, we remove first 5 and then a further 4 false alarms. In this example, the last 5 trials (after the second |) are all false alarms, so we assume that with a higher decision criterion these would all have been correct rejects; the number of hits is unchanged. The second point on the ROC curve thus has *N*_hit_=10, *N*_FA_=8 (and thus *P*_hit_=0.77, *P*_FA_=0.21). To remove a further 4 false alarms, we have to work our way up and remove the next 5 stimuli. We are thus left only with the 13 stimuli before the first |. The third point on the ROC curve thus has *N*_hit_=9, *N*_FA_=4 (and thus *P*_hit_=0.69, *P*_FA_=0.10).

5. Fitting coal tit data using order of decision

Coal tit data were analysed in the same way as human data using order of decision. Once again, selections were ignored after the number of false alarms had reached *N*_­tgt_. For example, bird *Ara* in the 3-day condition was searching for *N*_tgt_=8 seeds it had cached 3 days earlier in 8 out of 84 possible locations (*N*_lure_=76). 8 false alarms were reached after the bird had examined 13 locations, with the results FFH|FHHFHF|FHFF. The first point on the ROC curve thus has *N*_hit_=5, *N*_FA_=8, *N*_miss_=3, *N*_CR_=68, thus *P*_hit_=0.63, *P*_FA_=0.11. To generate the second point, we deselect the last 4 locations, removing 3 false alarms and 1 hit. Thus *N*_hit_=4, *N*_FA_=5; *P*_hit_=0.5, *P*_FA_=0.07. Finally, we deselect a further 6 locations, removing 3 false alarms and 3 hits. Thus *N*_hit_=1, *N*_FA_=2; *P*_hit_=0.13, *P*_FA_=0.03.

6. Supplementary Analysis: Examining for bias in our fitting procedure

The major conclusion of our paper is that whereas humans show Recollection of locations as well as Familiarity, birds show only Familiarity, with no evidence of Recollection. However, our data are undoubtedly noisy, with only 3 points estimated on the ROC curve, and only a small number of trials used. Furthermore, humans searched for 13 targets among 52 possible locations, whereas birds searched for 5-15 targets among 84 possible locations. Could this have caused an artefactual difference in Recollection, e.g. is R systematically underestimated when there are fewer targets? In this Appendix, we examine this.

### 6.1 Methods

We generated simulated data by assuming that N­_­tgt_ seeds are hidden in N_loc_=84 possible locations, and assuming values for *R* and *d’*.

To generate a particular set of simulated data, we first asked how many N_tgt_ seed locations were recollected (binomial probability distribution with probability *R* of recollecting each of N­_tgt_ locations); these N_rec_ sites were assumed to be visited first, as “hits”.

We then generated Familiarity signals for the remaining N­_tgt_-N_rec_ locations; these were random numbers drawn from a normal distribution with mean *d’* and SD 1. We then generated Familiarity signals for the N_loc_-N­_tgt_ lures; these were random numbers drawn from a normal distribution with mean 0 and SD 1. We had our simulated bird visit each location according to the order of the Familiarity signals, ranked from highest to lowest. The results were “hits” where the Familiarity signal came from a target location and “false alarms” where it came from a lure. As in the real data, we used only trials up to and including the trial where the total number of false alarms became equal to N­_tgt_.

We then fitted this simulated data in exactly the same way as for the original data.


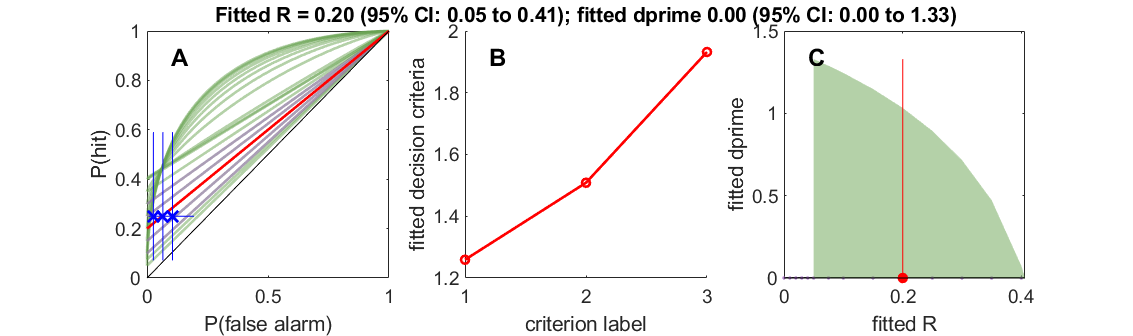


**Figure S4. Example fit to simulated data. In this example, the simulated bird had cached 8 seeds and had R=0.25, d’=1. On this simulated trial, the data used for fitting were HFHF|FFF|FFF and so the points on the ROC curve were (p_FA_, p_hit_) = (0.11, 0.25), (0.07,0.25) and (0.03,0.25).**

|

### 6.2 Results: Results for individual birds are noisy but unbiased. The number of seeds hidden has little effect either on noise or bias.

Figure S4 shows example results for a single simulated experiment, and Figure S5A shows the results for 100 different runs with the same parameters. It is clear that there is tremendous variability in the results of the individual runs, reflecting the sparse amount of data. Note also that there are only a limited number of fitted results that can be obtained, reflecting the limited number of possible sets of 3 points on the ROC curve which can be generated in this way. However, note that while there is noise, there is very little bias: The mean R and d’ of the fitted results (large +) are very close to the true value (small x). Critically, although some runs do result in estimated *R*=0, on average the fitted *R* is very close to the true value, 0.25. Thus, Figure S5A shows that even with a very small number of seeds and visits in an experiment, we can obtain an unbiased estimate of *R*. There is no artefact which causes *R* to be systematically underestimated.

Figure S5B shows the same thing for a naïve bird, with a lower d’ and R=0. Once again, the mean of the fitted results is close to the true value. R of course is overestimated, since our fitting constrained *R*≥0 and thus the mean fitted *R* cannot be 0. Figure S6 shows two further examples with 8 seeds.

**Figure S5. Recollection R and Familiarity d’ obtained by fitting simulated data. A) Simulated bird cached 8 seeds and had R=0.25, d’=1 (marked with a cross, x). Disks: results of fitting 100 different individual simulated experiments with this simulated bird. Large cross: mean ± SD of fitted results. Because many different runs resulted in the same 3 points on the ROC curve, the same fit values are also obtained many times. This is indicated by the saturation of each coloured disk. B) As (A) but for a simulated naïve bird with no Recollection. d’ is still >0, representing the birds’ tendency to cache in locations with particular properties.**

**Figure S6. More examples with 8 seeds, but different R and d’.**

Figures S7-S9 show results in the same format as Figure S6, but for the case where 4, 5 or 15 seeds were hidden. 15 was the maximum numbers hidden in our experimental data. 4 seeds is arguably too few. The fitting process often failed, and where it succeeded, it is striking that in every case, either the fitted d’ or the fitted R is zero. Overall, the fitted d’ is seriously underestimated; R, less so. However, since the main result of our paper is that bird d’ is larger than R, we decided to include the 6 trials where only 4 seeds were hidden (one retention interval in each of 3 different Retrieval birds; thus 3 Retrieval and 3 Naïve trials had N_tgt_=4). We did exclude a further 14 trials with N_tgt_<4.

However, there is relatively little difference between results for 5 and 15 seeds. With 5 seeds, the number of possible points on the ROC curve is still highly constrained, so there are relatively few distinct possible fitted values. However, the average value across several fits is close to the true value, i.e. there is little noise. Tripling the number of seeds hidden from 5 to 15 also has little effect on the noise: the standard error on the estimate of the mean is somewhat reduced, but not dramatically.

**Figure S7. As Figure S5, but with a simulated bird that cached 4 seeds. With so few seeds, there are fewer possible points on the ROC curve; this is why there are fewer distinct symbols, although the number of simulated experiments is still 100.**

**Figure S8. As Figure S7, but with a simulated bird that cached 5 seeds. With so few seeds, there are fewer possible points on the ROC curve; this is why there are fewer distinct symbols than in Figure 8, although the number of simulated experiments is still 100.**

**Figure S9. As Figure S7, but with a simulated bird that cached 15 seeds.**

### 6.3 Results: Including more decisions has little effect on results

In our analysis of experimental data, we transcribed the first 30 locations where the bird looked for seeds, but we only included visits until the bird had made N_­tgt_ false alarms (i.e. had looked in N­_tgt_ empty locations, where N_tgt_ was the number of seeds hidden in that experiment). In our simulations, we explored the effect of including more decisions (visits). Figure S10 shows an example which can be compared to Figure S4 : a different instantiation of the same random distribution but now including more decisions. Note that the 3 points on the ROC curve are now higher up, since more visits to locations necessarily produce more hits and more false alarms. Figure S11 shows the distribution of fitted estimates using enough visits to produce N_tgt_ false alarms vs using the first 30 visits (green), for three different sets of parameters. The variability (errorbars) is similar for both methods, and the difference in the mean values of the estimates is small compared to the variability, suggesting that there is no strong reason to prefer one over the other.


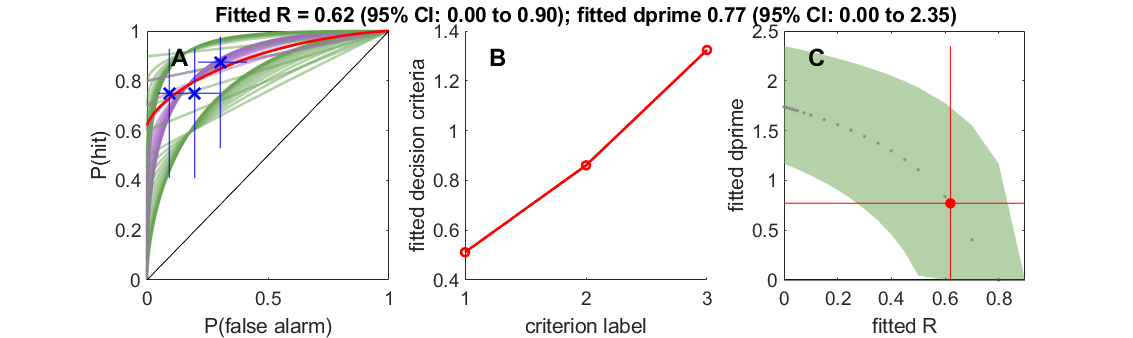


**Figure S10. Example fit to simulated data. In this example, as in Figure S4, the simulated bird had cached 8 seeds and had R=0.25, d’=1. However, now 30 visits were used for fitting. Accordingly, the 3 points tend to lie higher up on the ROC curve. On this simulated trial, the data used for fitting were HHHHHFHFFFFFF|FFFFFFFF|FHFFFFFFF and so the points on the ROC curve were (p_FA_, p_hit_) = (0.88, 0.30), (0.75,0.20) and (0.75,0.09).**

**Figure S11. Comparing the effect of using more vs fewer decisions. In panels A,B,C, the red/blue symbols are the same results shown in Figure A, Figure 5A, Figure B respectively. Green symbols are for simulations with the same N_tgt_, R and d’, but this time using all 30 trials. The true parameters are now shown with a black cross to indicate that these apply to both red and green.**

### 7. Supplementary Analysis: Statistical power

In our main paper, we concluded that birds showed no evidence of Recollection. Obviously, our limited statistical power constrains our ability to detect any difference that might in reality exist. We used our simulations to assess our statistical power. In these examples, we assume that the naïve birds must have *R*=0, and we compare our ability to discriminate this from a situation where the experienced birds have a non-zero *R*, taking into account the fact that we only have a small number of birds and that the error on individual estimates of *R* is very large. For simplicity, we assume here that all birds are identical and hid N_tgt_=8 seeds, so that we are drawing *N*_birds_ random variates from the same distribution: *N*_birds_ from the experienced distribution (*R*_exp_>0,d’_exp_≥0.3) and *N*_birds_ from the naïve distribution (*R*_nai_*=*0,*d’*_nai_=0.3). We then use the Wilcoxon paired signed-rank test to ask whether *R* differs between the experienced and naïve simulated birds. If *p*>0.05, then we have made a Type II error: we have failed to detect the difference which exists. We can repeat this process of drawing *N*_birds_ random variates from the memory and naïve distributions many times, in order to estimate our probability of making a Type II error, conventionally known as β. The results are shown in Figure S12 for two values of *N*_birds_ (panels A,B), for three values of *d’*_exp_ (colors) and for different *R*_exp_ (horizontal axis).

If Recollection does exist, it is likely to be greatest at shortest delays. Empirically, at the shortest delay of 1 day, we found R_exp_(1d) = 0.059, d’_exp_(1d) = 1.28, compared to R_nai_(1d)=0.052, d’_nai_(1d) = 0.25. A Wilcoxon paired signed-rank test indicates that the difference in d’ is highly significant (*p*=0.02) whereas the difference in R is not (*p*=0.74). However, we only have usable data from 8 pairs of birds at a delay of 1 day, since bird Sam hid only 4 seeds. As Figure S12A shows, our power is then extremely limited. Especially if d’_exp_(1d) really is large, we might well find that R_exp_(1d) is not significant, even it was in fact substantial.

However, if we pool the two shortest delays, we have 17 pairs of birds. Empirically, averaging across both delays, we then have R_exp_(1d,3d) = 0.15, d’_exp_(1d,3d) = 1.03 for the experienced birds, and R_nai_(1d,3d)=0.06, d’_nai_(1d,3d) = 0.33 for the naïve birds. Again, the difference in d’ is highly significant (*p*=0.0007) whereas the difference in R is not (*p*=0.96). Figure S12B indicates that for the empirical value of *R*_exp_, 0.15, 17 birds gives us close to 80% power. So, it is reasonably possible that the value of 0.15 for R is actually real, even though it was not significant.

However, we do have enough power to be >90% confident of detecting a real *R*_exp_ greater than around 0.3, close to the value found in our humans (Fig 3A of main paper). *R*=0.3 is roughly equivalent to *d*’=0.55, in the sense that both produce similar increases in performance (if *R* is fixed at 0, then an increase in Familiarity from *d*’=0 to *d*’=0.55 increases the area under the ROC curve by 0.15, the same as is produced by an increase in Recollection from *R*=0 to *R*=0.3 with *d*’ fixed at 0). In birds, the mean increase in *d’* was over 1, which would increase AUROC by 0.26, equivalent to *R*=0.52.

Thus, our conclusion that bird memory is overwhelmingly made up of increased Familiarity rather than Recollection is secure. We cannot rule out small increases in *R*, but we can be confident that these contributed much less to performance than the significant increases in *d*’.

**Figure S12. Probability of making a Type II error: i.e. finding no significant difference (at the α=0.05 level) between R in naïve and experienced birds, when R_nai_=0, d’_nai_=0.3 and R_exp_ ,d’_exp_ have the values shown on the x-axis/legend respectively. The results were obtained by running 10,000 Monte Carlo simulations in which we picked N_bird_ values drawn with replacement from 100 values of fitted R, obtained previously by running 100 simulations with d’_exp_ and R_exp_ , and a second set of N­_bird­_ values from 100 values of fitted R obtained from simulations with d’_nai_ and R_nai_ . For each Monte Carlo simulation, we ran a Wilcoxon rank sum test to see whether the two sets of N_bird_ R values differed significantly. The probability of Type II error, β, plotted in the graphs is the proportion of simulations in which the Wilcoxon significance p exceeded α. In all cases, N_tgt_=8, α=0.05. (A) N_bird_=9, corresponding to our power to detect Recollection at just one delay; (B) N_bird_=18, corresponding to our power to detect Recollection over two delays.**

### 8. Supplementary Analyses: Conclusions

As indicated by the large confidence intervals in Figure S2C, our fitting approach is necessarily imprecise, since it uses few trials searching for few seeds and estimates just 3 points on the ROC curve. However, it is not strongly biased, and in so far as it is biased, it tends to ascribe to Recollection what is in fact Familiarity, rather than the other way around. That is, *d’* tends to be under-estimated and *R* over-estimated.

In our experiments with real birds, we found that experience caching seeds was reflected in higher familiarity *d’* but not in higher Recollection *R*: *R* was the same in both naïve and experienced birds. Our simulation results suggest that the increase in *d’* was real, in fact perhaps underestimated. It also suggests that our analysis would have detected a comparable increase in *R*. We conclude that the lack of Recollection is highly unlikely to reflect some artefact of our parameter estimation which causes us not to detect Recollection when it is present. Rather, it likely represents the fact that memory genuinely does not increase Recollection in these birds.
